# Electro-Mechanical Ionic Channel Modeling for Uterine Contractions and Oxytocin Effect during Pregnancy

**DOI:** 10.3390/s19224898

**Published:** 2019-11-09

**Authors:** Yiqi Lin, Mengxue Zhang, Patricio S. La Rosa, James D. Wilson, Arye Nehorai

**Affiliations:** 1 The Preston M. Green Department of Electrical and Systems Engineering, Washington University in St. Louis, Saint Louis, MO 63130, USA; lin.yiqi@wustl.edu; 2 Decision Science, Bayer Company, Saint Louis, MO 63146, USA; mengxue.zhang.ext@bayer.com (M.Z.); patricio.larosa@bayer.com (P.S.L.R.); 3 Formerly at Graduate Institute of Technology, University of Arkansas at Little Rock, Little Rock, AR 72204, USA; jdwilson@ualr.edu

**Keywords:** uterine contractions, ionic channels, oxytocin, uterine cell force

## Abstract

Uterine contractions during normal pregnancy and preterm birth are an important physiological activity. Although the cause of preterm labor is usually unknown, preterm birth creates very serious health concerns in many cases. Therefore, understanding normal birth and predicting preterm birth can help both newborn babies and their families. In our previous work, we developed a multiscale dynamic electrophysiology model of uterine contractions. In this paper, we mainly focus on the cellular level and use electromyography (EMG) and cell force generation methods to construct a new ionic channel model and a corresponding mechanical force model. Specifically, the ionic channel model takes into consideration the knowledge of individual ionic channels, which include the electrochemical and bioelectrical characteristics of individual myocytes. We develop a new sodium channel and a new potassium channel based on the experimental data from the human myometrium and the average correlations are 0.9946 and 0.9945, respectively. The model is able to generate the single spike, plateau type and bursting type of action potentials. Moreover, we incorporate the effect of oxytocin on changing the properties of the L-type and T-type calcium channels and further influencing the output action potentials. In addition, we develop a mechanical force model based on the new ionic channel model that describes the detailed ionic dynamics. Our model produces cellular mechanical force that propagates to the tissue level. We illustrate the relationship between the cellular mechanical force and the intracellular ionic dynamics and discuss the relationship between the application of oxytocin and the output mechanical force. We also propose a simplified version of the model to enable large scale simulations using sensitivity analysis method. Our results show that the model is able to reproduce the bioelectrical and electromechanical characteristics of uterine contractions during pregnancy.

## 1. Introduction

We propose a forward model that is capable of reproducing the characteristics of uterine contractions. We introduce an ionic channel model, an oxytocin model and a mechanical force model in this work. Developing these models are important for understanding the mechanism of each individual ionic channel, as well as the whole mathematical expression of the system. The ionic model illustrates the natural biological properties of cells in the uterus and the application of oxytocin describes the expression of cells after clinical intervention. Thus, the results of this work can help us better understand the mechanisms of uterine cells, their force generation process and the results of applying oxytocin. Understanding these mechanisms is important in clinical practice regarding complications, such as preterm birth and abnormal uterine contractions, which can lead to significant medical and financial consequences [1,2,3,4].

Previous studies have indicated that myocytes in the uterus can generate different types of action potentials [5,6,7]. Therefore, research on action potential generation mechanisms is important to help us understand the biological and electrical activities of uterine contractions. Several mathematical models have been developed to describe the electrostatics of ionic channels [8,9,10]. These models basically illustrate the relationship between the input stimulation and the output membrane potential. However, the dimensions of most of these models are rather limited, so the model cannot fully express the biological properties of the ionic channels. Earlier studies developed mathematical models of action potential which included descriptions of many individual ionic channels [11,12,13]. However, the large number of individual channels made the whole model too complex and limited the further application of the 3D uterine contractions forward model. As an additional limitation, there is little work focusing on the influence of oxytocin on uterine contractions during pregnancy.

In our previous studies [14,15,16], we developed a multiscale model which takes into account the electrophysiological, anatomical and magnetomyographic knowledge [17,18] jointly at the cellular, tissue and organ levels during uterine contractions. In the previous model, we did not include specific ionic channels at the cellular level. Therefore, we develop several new ionic channels based on the potassium and sodium lab data from the human myometrium in this work. The ionic channels are derived from the Hodgkin and Huxley’s equations [19] and fitted by lab data on individual ionic currents with respect to various membrane potential values. Then, we replace these channels in Tong’s model [11] with our new channels which incorporate newly collected experimental data from human myometrium and the new ionic channel model is able to produce different types of action potentials, including the single spike, plateau-type, short burst and long burst action potentials reported in the previous studies [6,7]. Also, we know that the mechanical properties of the myometrium keep changing during gestation [20] and the study of cell force generation helps us better understand the mechanisms of uterine contractions. Thus, we introduce the mechanical force model which is derived from Hai and Murphy’s studies [21] on smooth muscle cells and the model is able to generate the cell mechanical force based on the newly developed ionic channel model.

Moreover, Mironnear [22] experimented on rat uterine strips and illustrated the change of inward current and action potential caused by the application of oxytocin and Nakao’s experiment [23] also found that the application of oxytocin increased the amplitude of the action potential without a significant change in the resting membrane potential. Here, we introduce a new oxytocin model to reproduce these experimentally observed phenomena. Our oxytocin model is developed as a clinical intervention to control the calcium current and influence the calcium concentration, action potential generation and force generation.

## 2. Materials and Methods

In this section, we propose several new uterine ionic channels. Individual ionic channels are simulated independently to fit the feature points derived from the lab data collected by our collaborator, Dr. Sarah England. The data collection process was conducted in conformance with the Declaration of Helsinki and were approved by the Institutional Review Board at the Washington University School of Medicine (approval no. 201108143), except for registration in a database. The data includes representative recordings from 4 patients and all of them signed written consent forms approved by the Washington University in St. Louis Internal Review Board. More detailed information regarding the lab experiment can be found in Reference [24].

We mainly focus on three ionic channels, which are sodium channel, potassium channel and calcium channel. These three channels play an important role in the depolarization and repolarization of the action potential generation process during uterine contractions. The whole ionic channel model is built by combining all the individual ionic channels according to Kirchhoff’s laws. We adopt Tong’s model [11] and replace several of its channels with our newly developed channels. Also, we consider the influence of oxytocin application on the ionic channels. Moreover, based on this new ionic channel model, a mechanical force model is introduced to describe the force characteristics during uterine contractions.

### 2.1. Sodium Channel

The sodium channel is largely inactivated at resting potential. When it is activated, a significant number of sodium ions move into the cell and it is responsible for the rising phase of the action potential during depolarization. In our model, the sodium channel is built by using raw lab data recorded by our collaborator [24]. The data came from sodium channels in the uterine cells of human myometrial tissue samples from the lower uterine segments. The membrane potential and the solutions were designed to minimize the contribution of the calcium and potassium channels. Here, the voltage-clamp sodium experimental data illustrate the electrical properties of sodium currents with respect to the membrane voltage values.

The model is fitted by Hodgkin and Huxley’s equations for the sodium channel [19]. The sodium channel is represented by the nonlinear conductance $g_{\mathrm{Na}}$, which depends on both time and voltage. To make this clear, we represent it by using the variable $x_{\mathrm{Na}}$. Here $x_{\mathrm{Na}}$ depends only on time and voltage [8]. Thus, the nonlinear sodium conductance is given by

(1) $$\begin{array}{c} g_{\mathrm{Na}}=x_{\mathrm{Na}}{\bar{g}}_{\mathrm{Na}}. \end{array}$$

We use *m* as an activation parameter and *h* as an inactivation parameter to describe the variable $x_{\mathrm{Na}}$. Here, the sodium channel current is given by

(2) $$\begin{array}{cc} I_{\mathrm{Na}} & =m^{3}h{\bar{g}}_{\mathrm{Na}}\left(V-E_{\mathrm{Na}}\right), \end{array}$$

(3) $$\begin{array}{cc} \frac{dm}{dt} & =\frac{m_{\infty }-m}{\tau_{m}}, \end{array}$$

(4) $$\begin{array}{cc} \frac{dh}{dt} & =\frac{h_{\infty }-h}{\tau_{h}}, \end{array}$$

where $m_{\infty }$ and $h_{\infty }$ are the steady state values of *m* and *h*, given a relatively long time; $\tau_{m}$ and $\tau_{h}$ are time constants which describe how fast *m* and *h* will approach the steady state; *V* is the applied voltage; and $E_{\mathrm{Na}}$ is the resting potential, which is a constant value given by the Goldman equation [25]. Here $m_{\infty }$, $h_{\infty }$ and the time constants are functions of the applied voltage *V* and follow the Boltzmann’s principle [26].

Accordingly, the ratio of the probabilities of a cell being in the membrane ($P_{1}$) and out of the membrane ($P_{2}$) follows an exponential function:

(5) $$\begin{array}{c} \frac{P_{1}}{P_{2}}=\exp\left(f\left(V\right)\right). \end{array}$$

For one particular cell, the sum of the probabilities should be one, that is, $P_{1}+P_{2}=1$, which gives us

(6) $$\begin{array}{c} P_{1}=\frac{1}{1+\exp(f(V))}. \end{array}$$

Thus, for the steady state, the values of the active parameter and inactive parameter can be described in the following form:

(7) $$\begin{array}{c} m_{\infty }=1/\left[1+\exp(f_{1}\left(V\right))\right], \end{array}$$

(8) $$\begin{array}{c} h_{\infty }=1/\left[1+\exp(f_{2}\left(V\right))\right], \end{array}$$

where $f_{1}\left(V\right)$ and $f_{2}\left(V\right)$ are functions that depend on the membrane potential. The functions can be expressed by the difference between the current membrane potential and the membrane potential at which 1/2 activation occurs [27]. We obtained the mathematical formulations of these equations from our lab data.

### 2.2. Potassium Channel

Potassium channels are classified by their different biological properties. The significant contributions come from the calcium activated potassium channel, voltage gated potassium channel, inward rectifier potassium channel, A-type transient potassium channel and background potassium leakage channel.

In our model, the potassium channel is built by using raw lab data recorded by our collaborator under the experimental conditions described in Reference [24]. The data came from potassium channels in the uterine cells of human myometrial tissue samples from the lower uterine segment. The patients were non-laboring women at term who had had Caesarean sections under spinal anesthesia. The recording focuses on potassium channel, so the membrane potential and the solutions were designed to minimize the contribution of the calcium and sodium channels.

Like the sodium channel, the potassium channel is represented by the nonlinear conductance $g_{K}$, which we represent by a variable $x_{K}$, which depends on time and voltage [8]. Thus, the nonlinear potassium conductance is given by

(9) $$\begin{array}{c} g_{K}=x_{K}{\bar{g}}_{K}, \end{array}$$

where $x_{K}$ is illustrated by the activation parameter *n* and inactivation parameter *q*. Hodgkin and Huxley proposed that four activation gates control the potassium channel [19]. From the lab data, we found that activation process is relatively faster than inactivation, so we use one inactivation gate to control the potassium channel. Therefore, the total current of the potassium channel is given by

(10) $$\begin{array}{c} I_{K}=x_{K}{\bar{g}}_{K}\left(V-E_{K}\right)=n^{4}q{\bar{g}}_{K}\left(V-E_{K}\right), \end{array}$$

where $E_{K}$ is the resting potential given by the Goldman equation. We describe the activation and inactivation gate variables by the following first order differential equations:

(11) $$\begin{array}{c} \frac{dn}{dt}=\frac{\left(n_{\infty }-n\right)}{\tau_{n}}, \end{array}$$

(12) $$\begin{array}{c} \frac{dq}{dt}=\frac{\left(q_{\infty }-n\right)}{\tau_{q}}, \end{array}$$

where $n_{\infty }$ and $q_{\infty }$ are the steady state values and $\tau_{n}$ and $\tau_{q}$ are time constants which describe how fast *n* and *q* will approach the steady state. According to Boltzmann’s principle, we describe the steady state values in the following equations:

(13) $$\begin{array}{c} n_{\infty }=1/\left[1+\exp(f_{3}\left(V\right))\right], \end{array}$$

(14) $$\begin{array}{c} q_{\infty }=1/\left[1+\exp(f_{4}\left(V\right))\right], \end{array}$$

where $f_{3}\left(V\right)$ and $f_{4}\left(V\right)$ are functions that depend on the membrane potential.

### 2.3. Calcium Channel

Calcium channels play an important role in the change of membrane potential. They activate on membrane depolarization and mediate calcium influx in response to action potentials and subthreshold depolarizing signals [28]. The two major calcium channels are the L-type and T-type. Here, we adopt Tong’s model [11] with regard to the L-type calcium channel and T-type calcium channel. The calcium currents are given by

(15) $$\begin{array}{cc} I_{\mathrm{CaL}} & =g_{\mathrm{CaL}}d^{2}f_{\mathrm{Ca}}\left(0.8f_{1}+0.2f_{2}\right)\left(V-E_{\mathrm{CaL}}\right), \end{array}$$

(16) $$\begin{array}{cc} I_{\mathrm{CaT}} & =g_{\mathrm{CaT}}b^{2}g\left(V-E_{\mathrm{CaT}}\right), \end{array}$$

where $g_{\mathrm{CaL}}$ and $g_{\mathrm{CaT}}$ are the calcium conductances; *d* and *b* are the activation gate variables; $f_{1}$, $f_{2}$ and *g* are the inactivation gate variables; $f_{\mathrm{Ca}}$ is the calcium inhibition; and $E_{\mathrm{CaL}}$ and $E_{\mathrm{CaT}}$ are the reversal potentials.

### 2.4. Oxytocin Model

Pharmacologic stimulation of uterine contractions and the use of agents to promote cervical ripening are common procedures in obstetric practice. Here, oxytocin is a most commonly used medication for these purposes [29] and the research on the oxytocin effect is an important topic for clinical applications. Our oxytocin model mimics the application of oxytocin as a clinical manual intervention. The influence of oxytocin is discussed with regard to the calcium concentration, membrane potential and the mechanical force model. In previous experiment studies, Mironnear [22] and Nakao [23] found that a low concentration of oxytocin will cause a slight depolarization of the resting potential and result in an increase of the amplitude of the action potential (from 5 mV to 10 mV). In a recent study [24], our collaborators showed that the activation of the oxytocin receptor inhibits SLO2.1 channels. This inhibition is linked to the opening of voltage-dependent calcium channels and the consequent increase of the intracellular calcium concentration (e.g., L-type and T-type). Buford’s work [30] introduced the oxytocin influence as a multiplier factor using the sigmoid response on the conductivity of the L-type calcium channel.

Based on these experimental results, our oxytocin model was developed to increase both the L-type and T-type inward calcium currents by using an amplifier variable which controls the conductivity of these calcium channels. The amplifier is given by the following equation:

(17) $$\begin{array}{cc} \mathrm{AM} & =\frac{0.37}{1+\exp-(\mathrm{oxy}-0.946)}+1, \end{array}$$

(18) $$\begin{array}{cc} {\tilde{g}}_{\mathrm{CaL}} & =\mathrm{AM}\times g_{\mathrm{CaL}}, \end{array}$$

(19) $$\begin{array}{cc} {\tilde{g}}_{\mathrm{CaT}} & =\mathrm{AM}\times g_{\mathrm{CaT}}, \end{array}$$

where oxy represents the concentration of oxytocin applied in the experiment. The new L-type calcium conductance, ${\tilde{g}}_{\mathrm{CaL}}$ and T-type calcium conductance, ${\tilde{g}}_{\mathrm{CaT}}$, were calculated by the product of the amplifier, AM and the original calcium conductances, $g_{\mathrm{CaL}}$ and $g_{\mathrm{CaT}}$. Note that as we show in our simplified model in Section 3.5, both L-type and T-type calcium channels are key elements to control the action potential.

In Equation (17), the amplifier is described using a sigmoid function [31] which can express the biological and electrical properties of the oxytocin application and meets the experimental results. The parameters are fitted by lab data from Nakao [23]. Also, the application of oxytocin on the calcium channels reveals the change of uterine mechanical force since calcium channels are the most important channels related to uterine mechanical force.

### 2.5. Force Model

We describe the uterine contraction force model based on the calcium concentration, under the assumption that calcium-dependent myosin phosphorylation is the only postulated regulatory mechanism that generates the cross-bridge cycling in uterine smooth muscle [21]. Here we adopt a cross-bridge model that describes the kinetics of myosin phosphorylation from Hai and Murphy [21] and the model can be described by four differential equations:

(20) $$\begin{array}{cc} \frac{d\left[M\right]}{dt} & =-K_{1}\left[M\right]+K_{2}\left[\mathrm{Mp}\right]+K_{7}\left[\mathrm{AM}\right], \end{array}$$

(21) $$\begin{array}{cc} \frac{d\left[\mathrm{Mp}\right]}{dt} & =K_{4}\left[\mathrm{AMp}\right]+K_{1}\left[M\right]-\left(K_{2}+K_{3}\right)\left[\mathrm{Mp}\right], \end{array}$$

(22) $$\begin{array}{cc} \frac{d\left[\mathrm{AMp}\right]}{dt} & =K_{3}\left[\mathrm{Mp}\right]+K_{6}\left[\mathrm{AM}\right]-\left(K_{4}+K_{5}\right)\left[\mathrm{AMp}\right], \end{array}$$

(23) $$\begin{array}{cc} \frac{d\left[\mathrm{AM}\right]}{dt} & =K_{5}\left[\mathrm{AMp}\right]-\left(K_{7}+K_{6}\right)\left[\mathrm{AM}\right], \end{array}$$

where M is the detached dephosphorylated cross bridge, MP is the detached phosphorylated cross bridge, AMp is the attached phosphorylated cross bridge, AM is the attached dephosphorylated cross bridge and $K_{1}$-$K_{7}$ are the rate constants.

## 3. Results and Discussion

### 3.1. Sodium Channel

By using voltage clamp raw lab data, the activation and inactivation gate variables are fitted by the following equations:

(24) $$\begin{array}{cc} f_{1}\left(V\right) & =-\frac{V+20.3}{6.2}, \end{array}$$

(25) $$\begin{array}{cc} f_{2}\left(V\right) & =\frac{V+46.2}{7.2}, \end{array}$$

(26) $$\begin{array}{cc} m_{\infty } & =1/\left[1+\exp\left(-\frac{V+20.3}{6.2}\right)\right], \end{array}$$

(27) $$\begin{array}{cc} h_{\infty } & =1/\left[1+\exp\left(\frac{V+46.2}{7.2}\right)\right]. \end{array}$$

**Figure 1:**
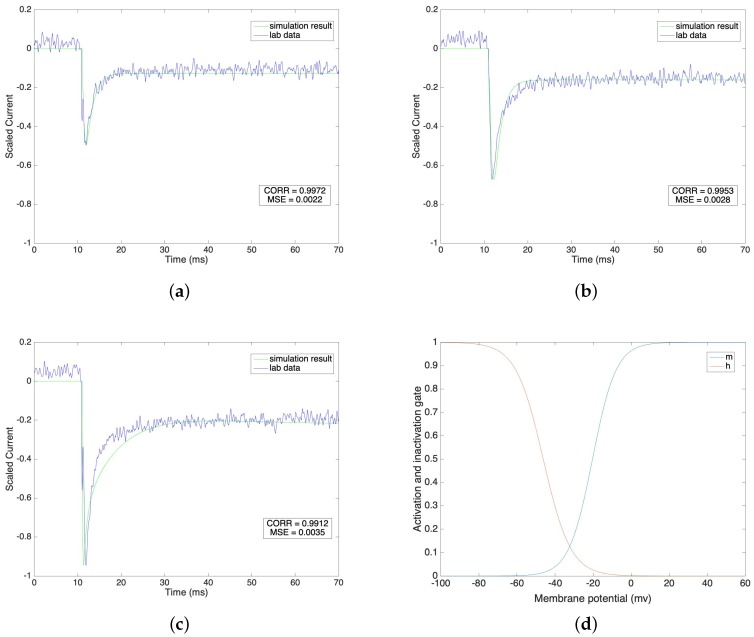
(**a**–**c**) Sodium current under different voltage clamp values(−40 mV, −20 mV and 0 mV). (**d**) Steady state of activation and inactivation gates dependent on membrane potential.

We compare the fitted model of sodium currents with the raw data in Figure 1a–c under different voltage clamp values from −40 mV to 0 mV, where the steps equal 20 mV. We normalize the current value on the vertical axis in order to have a direct view of the result under each voltage clamp condition.

Figure 1a–c indicate that the fitted sodium currents effectively trace the raw data after the stimulus. The stimulus is given at 11 ms and the lab data shows that the value of the sodium current reaches its peak value very quickly, undergoes a fast initial decay and then slowly decays further to a steady state. We mimic the lab data by setting the time constant for the activation gate, $\tau_{m}$, to a low level compared with the high level of the time constant for the inactivation gate, $\tau_{h}$. Under different voltage clamp values, the activation and inactivation processes of the sodium channel model accurately fit the lab data. We calculate the correlations and the corresponding mean square errors of our simulation results with the raw data at the same time steps. The correlation results are 0.9972, 0.9953 and 0.9912 and the corresponding mean square errors are 0.0022, 0.0028 and 0.0035 under voltage clamp values of −40 mV, −20 mV and 0 mV, respectively. This result shows that our simulation models are reliable and consistently follow the same pattern as the raw lab data. The steady state values of the activation gate and inactivation gate are compared in Figure 1d.

### 3.2. Potassium Channel

We fitted the potassium channel model with the lab data under the following conditions. The recording captured the potassium channel of human myometrial smooth muscle cells (hMSMCs), so calcium and sodium channels were inhibited. The experimental solution contained symmetrical potassium under 160 mM KCl, 80 mM NaCl, 10 mM Hepes and 10 mM MES and the pH value was 7.2.

Here, given the bio-electrical properties, the activation and inactivation gate variables are calculated by Boltzmann’s principle [26]:

(28) $$\begin{array}{cc} f_{3}\left(V\right) & =-\frac{V+13}{13}, \end{array}$$

(29) $$\begin{array}{cc} f_{4}\left(V\right) & =\frac{V+38}{6}, \end{array}$$

(30) $$\begin{array}{cc} n_{\infty } & =1/\left[1+\exp\left(-\frac{V+13}{13}\right)\right], \end{array}$$

(31) $$\begin{array}{cc} q_{\infty } & =1/\left[1+\exp\left(\frac{V+38}{6}\right)\right]. \end{array}$$

**Figure 2:**
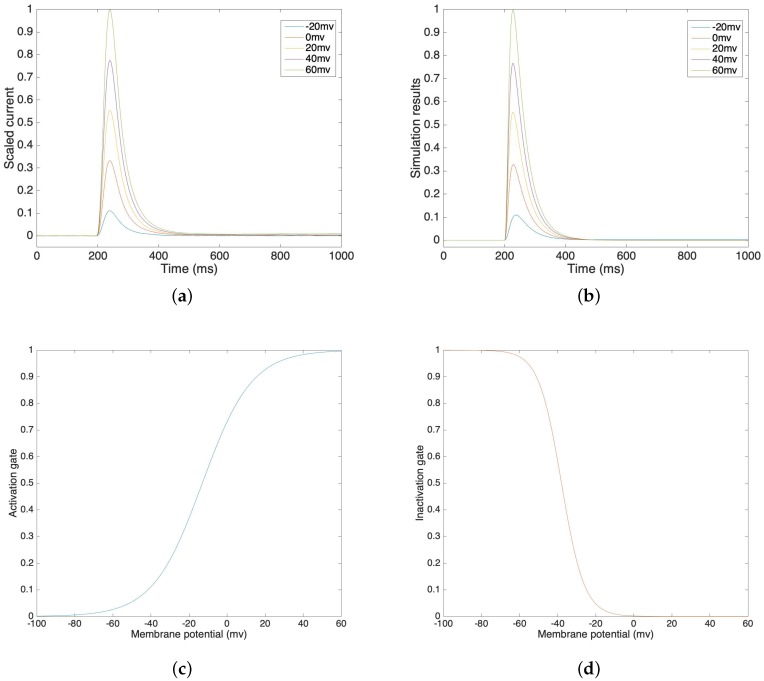
(**a**) Voltage clamp potassium channel lab data at voltage steps of −20 mV to 60 mV. (**b**) Voltage clamp potassium channel simulation result at voltage steps of −20 mV to 60 mV. (**c**) Simulated activation gate variable. (**d**) Simulated inactivation gate variable.

Figure 2a illustrates a lab recording of voltage clamp potassium current from −20 mV to 60 mV, in 20 mV steps. Figure 2b illustrates the simulation results of potassium current under different voltage clamp values. In order to compare our simulation results in Figure 2b and the lab data in Figure 2a, we compute the correlations and the corresponding mean square errors of these two values at the same time steps. The average correlation is 0.9945 and the average mean square error is 0.0039 under voltage clamp values from −20 mV to 60 mV. This result demonstrates that the simulation models accurately trace the lab data and that they are highly correlated. We can see that the inactivation process is relatively slower than the activation process, which also fits our assumption. Figure 2c,d show the activation gate variable and the inactivation gate variable, which depend on the membrane potential.

### 3.3. Oxytocin Model

**Figure 3:**
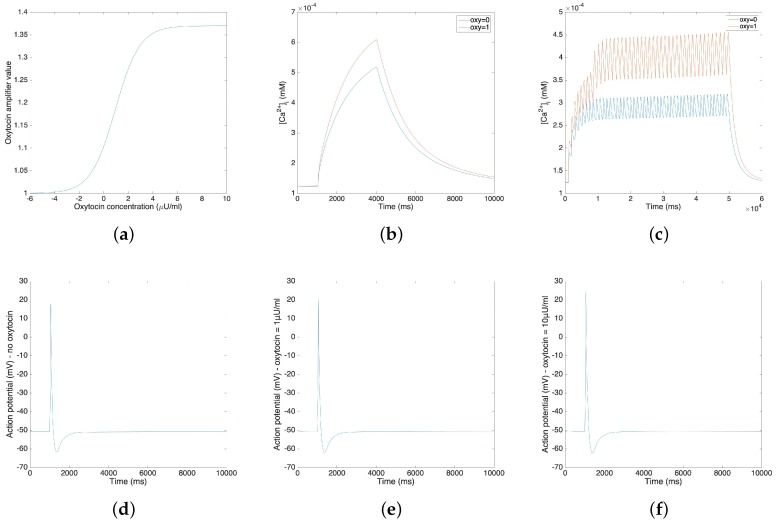
(**a**) Oxytocin amplifier value with respect to the concentration of oxytocin. (**b**) Change of calcium concentration of plateau type action potential under the application of 1 µU/mL oxytocin. (**c**) Change of calcium concentration of long burst type action potential under the application of 1 µU/mL oxytocin. (**d–f**) Action potentials under the application of different concentrations of oxytocin (no oxytocin, 1 µU/mL oxytocin and 10µU/mL oxytocin).

The oxytocin model is fitted by the amplitude change of the action potential in response to different concentrations of oxytocin. We adopt Tong’s model [11] in the following steps and replace the sodium and potassium channel $K_{1}$ with our new channels. Figure 3a illustrates the relationship between the value of the amplifier variable and the concentration of the applied oxytocin. Figure 3b,c compare the change of calcium concentration before and after the application of 1 µU/mL oxytocin, showing both the plateau type action potential and long burst type action potential. Figure 3b,c show that the application of 1 µU/mL oxytocin increases the peak of the calcium current by 20–40%. A comparable simulation result was reported for a sodium free solution by Mironneau [22].

Figure 3d–f show the influence of oxytocin on a single spike of the action potential. Figure 3d is the original action potential without the application of oxytocin; Figure 3e,f illustrate the action potential under the application of 1 µU/mL oxytocin and 10 µU/mL oxytocin.

Figure 3b–f show that the application of oxytocin will increase both the calcium concentration and the amplitude of the action potential. However, the resting membrane potential does not show a significant change. Also, the application of 1 µU/mL oxytocin increases the amplitude of the action potential by 3.79 mV and the application of 10 µU/mL oxytocin increases the amplitude of action potential by 6.65 mV. These simulation results fit the experimental data [23].

**Figure 4:**
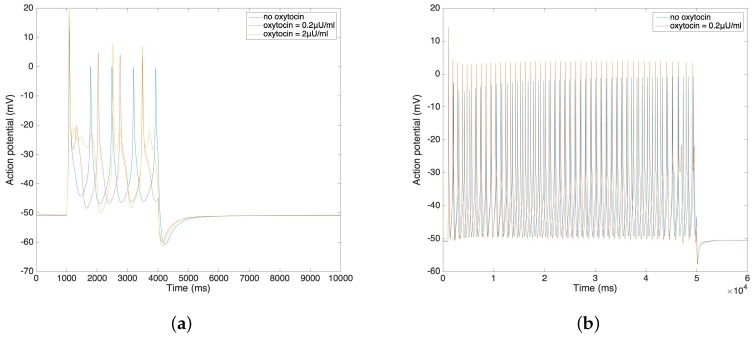
(**a**) Short burst action potential under the application of oxytocin at different concentrations. (**b**) Long burst action potential under the application of oxytocin.

In Figure 4a,b, we further compare the change of action potential with the application of different amounts of oxytocin. The simulation result from Figure 4a illustrates that the amplitude of the action potential increases by 5–10 mV after the application of 0.2 µU/mL and 2 µU/mL oxytocin. We observe that the time duration of the action potential increases with the increment of the oxytocin concentration, which is consistent with data from Mironneau [22] and Nakao [23]. Figure 4b shows the result of applied oxytocin on the long bursting action potential. The amplitude of each spike increases and the time duration between each spike decreases. This simulation result also fits Nakao’s experimental results [23].

### 3.4. Uterine Cell Force Model

The uterine cell mechanical force is represented by the uterine cell stress with a correlation coefficient. Based on the experiment by Hai and Murphy [21], the smooth muscle stress equals the sum of the attached phosphorylated cross bridge and the attached dephosphorylated cross bridge (AM+AMp). The force development is normalized by a correlation coefficient to fit the experimental results [32]. Also, our model shows how the application of oxytocin affects force development.

**Figure 5:**
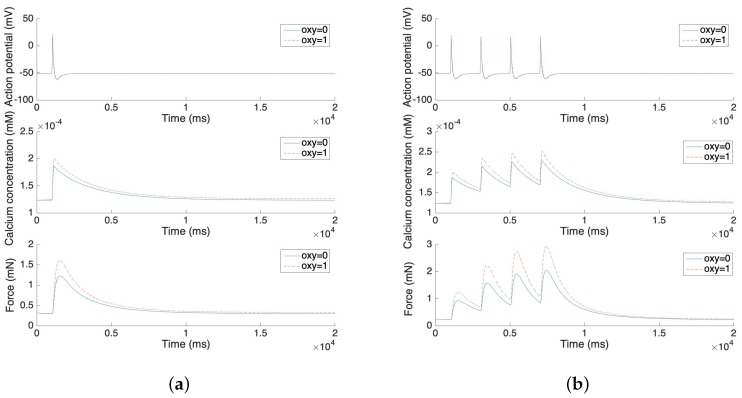
(**a**) Force development and oxytocin application of single spike action potential. (**b**) Force development and oxytocin application of four consecutive single spike action potentials.

Figure 5 illustrates the force development of the single spike and multiple spike action potentials. The solid line represents the model without oxytocin and the dashed line represents the model under the application of 1 µU/mL oxytocin. The experimental results from Burdyga [32] are replicated by these force development simulation results. After the stimulus, both the calcium concentration and the force increases rapidly. However, it takes more than 10 s for the force to return to the steady state. Also, the application of oxytocin increases the maximum of the calcium concentration by 10–20% and further increases the force by 25–50%. These phenomena also fit experimental results in the literature [33,34].

### 3.5. Model Simplification To Enable Large Scale Simulations

In order to run large scale simulations (e.g., our previous work in the 3D model [14,15]), we simplified the ionic channel model to lower the dimension and reduce the number of variables. Here we use Rihana’s sensitivity analysis method [35] to sort the variables and then complete the stability analysis. The analysis starts at an unstable steady state. Then, we add a perturbation on each element of the main diagonal of the Jacobian matrix and record the smallest absolute value of the perturbation which stabilizes the linearized system.

**Figure 6:**
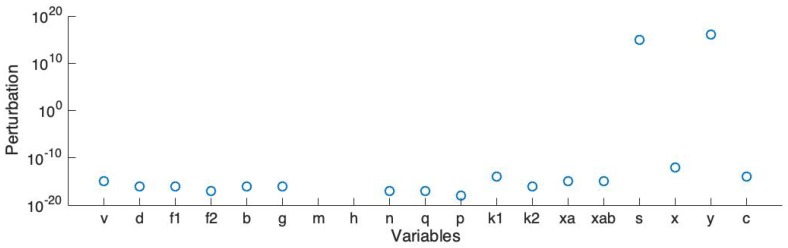
Stability analysis: smallest absolute values of perturbation on each variable which stabilize the system.

Figure 6 shows the smallest absolute values of perturbation on each variable which make all the eigenvalues of the Jacobian matrix have a negative real part. Here *v* represents the action potential; *d*, f1 and f2 represent the L-type calcium current; *b* and *g* represent the T-type calcium current; *m* and *h* represent the sodium current; *y* represents the hyperpolarisation-activated current; *n* and *q* represent the voltage-dependent potassium current $K_{1}$; *p*, k1 and k2 represent the voltage-dependent potassium current $K_{2}$; *s* and *x* represent the transient potassium current; xa and xab represent the calcium-activation potassium current; and *c* represents the calcium-activated chloride current. Also, the back current, sodium potassium pump current and sodium calcium exchanger current are dependent on *v*; the non-selective cation current is dependent on both *v* and the calcium concentration.

The linearized model becomes stable if the perturbation is added on each element of the main diagonal of the Jacobian matrix. Based on this result, we select the variable pairs for which the corresponding perturbations are comparatively smaller than the others. We sort all the currents and choose the first four currents: the L-type calcium current ($I_{\mathrm{CaL}}$), the T-type calcium current ($I_{\mathrm{CaT}}$), the voltage-dependent potassium current $K_{1}$ ($I_{K1}$) and the voltage-dependent potassium current $K_{2}$ ($I_{K1}$). Also, we choose the non-selective cation current ($I_{\mathrm{NSCC}}$), which has the largest magnitude. Therefore, there are 5 channels, represented by 13 variables, included in the simplified model. The simplified ionic channel model is described in the following equations:

(32) $$\begin{array}{cc} I_{\mathrm{sim}} & =I_{\mathrm{CaL}}+I_{\mathrm{CaT}}+I_{K1}+I_{K2}+I_{\mathrm{NSCC}}, \end{array}$$

(33) $$\begin{array}{cc} C_{m}\frac{dV_{\mathrm{sim}}}{dt} & =-I_{\mathrm{sim}}, \end{array}$$

(34) $$\begin{array}{cc} V_{\mathrm{ap}} & =V_{\mathrm{sim}}+V_{b} \end{array}$$

where $I_{\mathrm{sim}}$ is the total ionic current, which is a sum of the five chosen currents; $C_{m}$ is membrane capacitance; $V_{\mathrm{sim}}$ is the membrane potential simulated by the five chosen currents; $V_{b}$ is the background membrane potential, which is a constant value to offset the action potential; $V_{\mathrm{ap}}$ is the simulated output action potential. Note that $I_{\mathrm{CaL}}$ and $I_{\mathrm{CaT}}$ can be modulated by the oxytocin model in Equation (17). The following results do not include the application of oxytocin.

**Figure 7:**
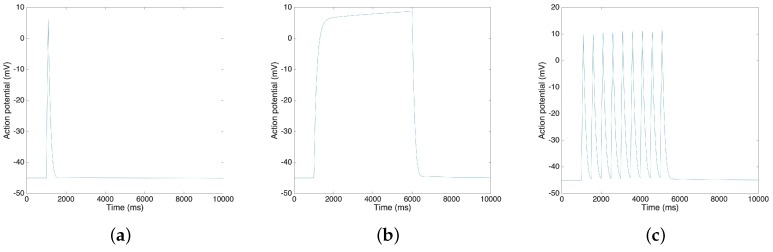
MATLAB simulation results of different types of action potential: (**a**) Single spike; (**b**) Plateau; (**c**) Bursting.

Using the Xppaut software [36], we find that the simplified model is stable, and we are able to calculate the new steady state of the variables. Figure 7 shows the result of action potentials by MATLAB simulation. By using the MATLAB, we generate various types (single spike, plateau and bursting) of action potentials, and to reproduce the electrical activities of the uterine contraction. In particular, as reported in Reference [5], the resting potential is approximately −45 mV and the time duration of the plateau type action potential is approximately 5 s. Also, the range of the action potential is approximately 55 mV as reported in Reference [37]. We observe that the resting potential of our model is −44.9 mV, that the ranges are around 55 mV in three cases and that the time duration of the plateau type action potential is 5 s, values that are in fair agreement with the reported values in the literature [5,37].

## 4. Conclusions

We developed a ionic channel model, an oxytocin model and a corresponding mechanical force model of uterine contractions during pregnancy. Our approach incorporated electromyography (EMG) and cell force generation methods to compute the electrical and mechanical properties of the uterus. The newly developed ionic channel model considered the electrochemical and bioelectrical characteristics of individual myocytes, which are important biosensors in myometrium. The mechanical model was based on the ionic channel model and the cellular mechanical force was introduced in this model. The project also took into consideration the influence of the application of oxytocin on both the ionic channel model and the mechanical force model. Moreover, we simplified our ionic channel model and our simplified model reproduces the biological and electrical activities of the uterine contractions. In general, our three models were able to mimic the change and propagation of electrical field patterns and uterus mechanical force during uterine contractions. They also demonstrated the influence of oxytocin on the action potential, cell mechanical force and uterine contractions. In conclusion, these models were capable of reproducing the characteristics of uterine contractions during pregnancy.

For future work, we will improve the accuracy of our ionic channel model by using more lab data, especially by using data of multiple ionic channels on the human uterus. We will incorporate this model into our multiscale 3D human uterus model [14,15] to study the propagation of ionic currents on the uterine surface, their response to changes in oxytocin, and its effect on uterine pressure. Also, we plan to build a prediction model that can estimate human delivery time and help diagnose preterm labor.
